# Pro- and Antifluoride Use Messages on YouTube in Japan: Content Analysis

**DOI:** 10.2196/82265

**Published:** 2025-12-29

**Authors:** Hikari Sophia Nagao, Tsuyoshi Okuhara, Hiroe Suzuki-Chiba, Hiroko Okada, Takahiro Kiuchi

**Affiliations:** 1 Department of Health Communication, Graduate School of Medicine, The University of Tokyo, 7-3-1 Hongo, Bunkyo-ku, Tokyo, 113-8655, Japan, 81 3-5800-6549, 81 3-5689-0726; 2 Department of Health Communication, School of Public Health, Graduate School of Medicine, The University of Tokyo, Tokyo, Japan

**Keywords:** caries prevention, fluoride, health communication, misinformation, oral health, social media

## Abstract

**Background:**

Dental caries is one of the most prevalent chronic conditions globally. In Japan, fluoride application—mainly via toothpaste, mouth rinses, and professional treatments—is a key preventive measure, as community water fluoridation is not implemented. Despite its proven effectiveness, fluoride use faces opposition from certain groups citing potential health risks. Social media platforms, especially YouTube, have become major sources of health information, but also facilitate the spread of misinformation, which may influence public perceptions and behaviors toward fluoride use.

**Objective:**

This study aimed to analyze YouTube videos addressing fluoride use for caries prevention, focusing on the types of information presented and comparing the messages shared by proponents and opponents of fluoride use.

**Methods:**

A comprehensive search was conducted on YouTube using fluoride-related keywords in Japanese. The top 50 videos for each keyword were screened, and after excluding irrelevant or duplicate content, 86 videos were analyzed. Videos were categorized as proponent (“pro”), opponent (“anti”), or others. The sources, intended audiences, and content themes were assessed. Interrater reliability was confirmed using the Cohen κ coefficient.

**Results:**

Of the 86 analyzed videos, 58% (n=50) were categorized as “pro,” 22% (n=19) as “anti,” and 20% (n=17) as others. Proponent videos, mainly produced by dental professionals, emphasized scientific evidence, such as the mechanism of fluoride in preventing caries and guideline-based recommendations. Opponent videos, largely uploaded by laypersons, highlighted potential dangers of fluoride, including health risks and additives, and frequently promoted fluoride-free products. Opponent videos had higher daily viewership and engagement than proponent videos.

**Conclusions:**

Anti-fluoride content on YouTube appears to reach broader audiences than expert-generated profluoride videos. Opponent messages tend to use emotionally charged communication, whereas proponents focus on scientific information. These differences in style may influence public perceptions of fluoride use. Public health professionals should develop engaging and accessible communication strategies to counter misinformation and promote evidence-based practices.

## Introduction

Dental caries is a pathological condition characterized by the progressive degradation of the hard tissues of the teeth instigated by acid production from bacterial metabolism. This process begins in the enamel and cementum, advances toward the dentin, and culminates in severe structural damage [1]. Dental caries remains a significant global public health challenge. According to the Global Burden of Disease Study 2021, major oral conditions such as untreated dental caries of permanent teeth, severe periodontitis, and edentulism affected an estimated 3.69 billion people worldwide in 2021 [2]. Among these conditions, untreated dental caries of permanent teeth was the single most prevalent specific condition [2]. Despite decades of preventive efforts, the global burden of oral conditions has changed little over the past 30 years [2]. These conditions exert profound adverse effects on the quality of life and socioeconomic systems [3].

Fluoride application is recognized as one of the most effective evidence-based measures for preventing dental caries [1]. Fluoride is widely recommended as a core strategy for caries prevention on the basis of long-standing evidence that, at recommended concentrations, its benefits outweigh potential risks [1]. The use of fluoride for the prevention of dental caries is broadly categorized into systemic and topical applications. In many countries, systemic fluoride application is implemented through fluoridation, a public health strategy involving the addition of fluoride to tap water supplies for consumption as drinking water [4]. Topical fluoride application constitutes the primary preventive approach, including the use of fluoride-containing toothpaste and mouth rinses, alongside professional fluoride treatments applied directly to tooth surfaces [5]. In Japan, approximately one-third of the population is estimated to have untreated caries, particularly among older adults who retain more natural teeth later in life [6]. While community water fluoridation is not implemented in Japan [7], prevention largely depends on individual behaviors [5]. In addition, naturally occurring fluoride in tap water varies geographically within Japan, and higher fluoride levels have been associated with a lower prevalence of reported treatment for dental caries in children [8].

Despite extensive evidence supporting its safety and efficacy, fluoride use faces opposition from groups citing potential health risks [9-11]. While excessive fluoride ingestion is a risk factor for fluorosis [12], adverse effects are dose-dependent and unlikely to occur under typical recommended use conditions [13]. In some cases, opposition to fluoride is embedded in broader holistic positions that also reject vaccines and certain dental materials [14,15]. Historically, the patriarchal medical system in Japan has granted physicians significant authority over treatment decisions [16]. Consequently, dentists’ opinions may play a critical role in shaping the course of treatment.

Recently, social media has become a primary source of health information, but it also facilitates the rapid spread of misinformation [17,18]. YouTube, one of the leading social media platforms in Japan with over 90% use among younger generations [19], is a key arena for this conflict. Sensational, simple, and emotionally charged content can elicit strong reactions and sharing [20,21], whereas information attributed to trusted experts is more credible and persuasive in encouraging health-related behaviors [22-24]. Similar dynamics have been observed in other public health topics, such as vaccine hesitancy, where misinformation can shape public perceptions and behaviors [25-27].

Previous studies have shown that anti-fluoride messages on social media are sometimes more prevalent than profluoride messages [28], and are often linked to the promotion of fluoride-free products [29]. On YouTube, professionally produced profluoride videos have lower viewership than popular antifluoride videos [30]. On Instagram, findings are mixed; while one study found more profluoride posts with widespread misinformation [31], other analyses identified more antifluoride content centered on conspiracy theories, health risks, and the promotion of natural or vegan fluoride-free products [32,33]. On Twitter (subsequently rebranded X), professional fluoride treatments are viewed positively, but discussions on water fluoridation are polarized [34]. The fluoride-free trend is largely driven by growing public interest in natural lifestyles [35].

However, to the best of our knowledge, very little research has analyzed the content of pro- and antifluoride messages on YouTube in Japan. This research gap is particularly important in Japan, where there is no community water fluoridation [7], and caries prevention depends largely on individual behaviors and parental consent. Parental opposition to fluoride is a barrier to fluoride treatments [36] and is linked to vaccine refusal [37], which may increase disease burden and health care costs [38]. As YouTube is a major platform for health information, this study aimed to evaluate the content of pro- and antifluoride YouTube videos on caries prevention.

The research question is outlined as follows: What specific content is disseminated by the proponents and opponents of fluoride use on YouTube videos?

## Methods

### Study Design

This study used quantitative content analysis to systematically review YouTube videos on fluoride use.

A total of 86 videos were deemed eligible for analysis. These videos were categorized as follows: 50 (58%) videos advocating fluoride use, 19 (22%) opposing fluoride use, and 17 (20%) presenting mixed or neutral viewpoints.

### Search Strategy: Data Collection

We conducted internet searches on October 15, 2023, using keyword combinations input in Japanese text (Multimedia Appendix 1) with methodologies used in previous research analyzing YouTube videos on health-related information [39]. A comprehensive search was conducted on YouTube using a range of relevant keywords, including “fluoride,” as well as additional terms identified via Google Trends, such as the following:

“fluoride AND coating,” “fluoride AND processing,” “dentist AND fluoride,” “fluoride AND coat,” “fluoride AND toothpaste,” “fluoride AND pan coating,” “fluoride AND resin,” “fluoride AND danger,” “fluoride AND rinse,” and “fluoride.”

The search results were sorted based on “view count,” and the top 50 videos for each keyword were selected for analysis.

To minimize bias, all searches were performed using Google Chrome in incognito mode to prevent the influence of personal browsing history or tailored search results. After each search, we closed the browser and reopened it in incognito mode, and no personal Google accounts were used. For each search formula, the top 50 results were reviewed, and duplicate results were excluded. Results concerning no association with fluoride use for caries prevention (eg, content about Teflon-coated cookware), videos shorter than 1 minute in length, or videos presented in non-Japanese languages were excluded. TV news clips were also excluded to ensure a homogeneous sample by focusing on content originally created for the YouTube platform, rather than repurposed broadcast media.

### Data Extraction

#### Basic Characteristics

The following basic information was collected from each video: the number of days since upload, total view count, number of likes, source, intended audience, and content. To assess viewership trends, the number of views was divided by the number of days since upload to calculate the average number of views per day.

We identified the source of each video based on the YouTube channel that uploaded it and categorized it into 6 groups: dentist or dental hygienist, local government, company, dental association, layperson, and unknown.

#### Content Characteristics

The first author (HSN) evaluated the intended audience for the video and categorized it as either the general public or dental hygienists. HSN reviewed the content of each video and categorized it into one of the 3 groups: pro, anti, or others based on the stance toward fluoride use for caries prevention. Multimedia Appendix 2 outlines the definitions of these categories. Subsequently, HSN categorized the videos according to their source and assessed the prevalence of advantages and disadvantages. The categorization according to their source was conducted only for “pro” and “anti” opinions, and “others” were not included.

The content of the videos was subsequently analyzed according to their source, with a focus on identifying trends and patterns in the stance toward fluoride use for caries prevention. HSN coded the videos in terms of their content characteristics and extracted fundamental information about the videos, including their main characters and opinions.

#### Themes and Codes

HSN watched the extracted videos in detail and inductively assigned categories and themes to the extracted descriptions of opinions on fluoride. The analysis generating themes was conducted only for “pro” and “anti” opinions, and “others” were not included. Multiple categories were selected for videos featuring two or more categories.

HSN extracted notable descriptions from each video and entered them into a Microsoft Excel spreadsheet. For each video, HSN generated a rephrasing of each notable description by briefly summarizing it. HSN then generated a theme name that summarized the rephrased descriptions and defined the themes. HSN used this process for all the videos. Theme names and definitions were updated during analysis. HSN outlined the final categories and themes, accompanied by the corresponding numbers and percentages of videos per category and theme.

#### Intercoder Reliability

To determine intercoder reliability, 2 independent coders (HSN and HS) assessed approximately 26% (23/86) videos in the final dataset that were selected at random. Using the codes and definitions of themes created by the first author (HSN), the third author (HS) evaluated (1) the themes ascribed to the videos and (2) the codes to which the themes were assigned. HS was instructed on the criteria for theme extraction and categorization for each theme during a meeting with HSN that lasted for approximately 1 hour. The discussion between HSN and HS was based on a coding manual. In the event of a discrepancy between the evaluations of HSN and HS, we engaged in a discussion to reach a consensus on the final theme and classification.

### Statistical Analysis

Descriptive statistics, including means, ranges, and percentages, were computed for each variable in the basic video information dataset. This analysis provided a comprehensive overview of metrics, such as the average number of views per day, total view count, and distribution of video sources and content codes. To assess the reliability of the coding, the Cohen κ coefficient was used to evaluate the interrater agreement. The analysis was conducted using R for Mac (version 4.4.2; R Foundation for Statistical Computing).

### Ethical Considerations

This study did not involve human or animal participants, and all data were collected from publicly available web-based sources. To maintain anonymity, all identifying information, such as YouTube user IDs, was deidentified during data reporting. This study was granted an exemption from ethical approval by the ethics review committee of the Graduate School of Medicine, University of Tokyo.

## Results

**Figure 1.**
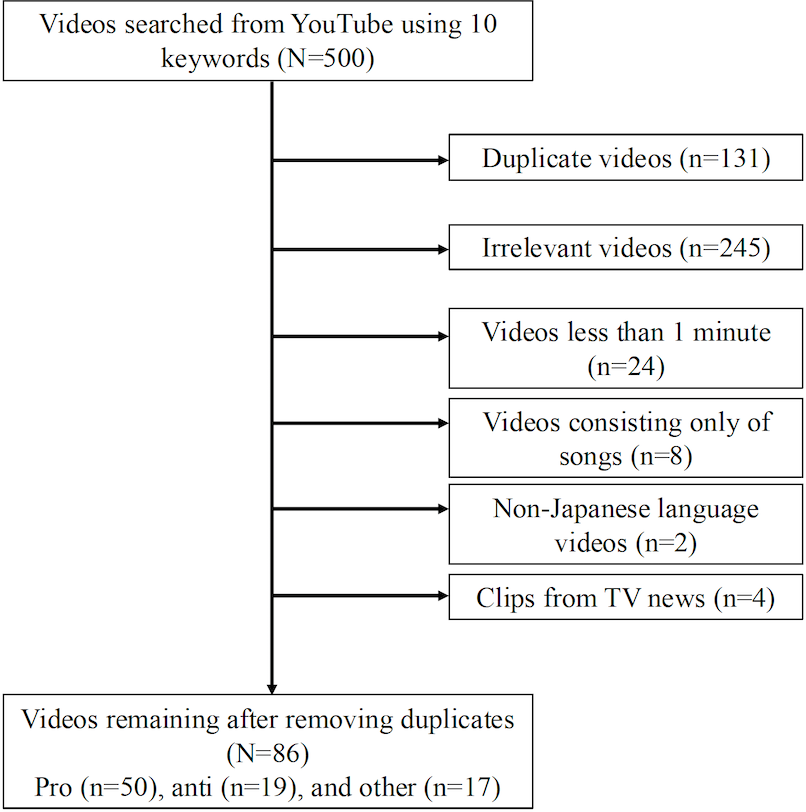
Search process for YouTube videos on fluoride use.

Table 1 summarizes the video characteristics. The average values and ranges (minimum to maximum) of the videos are listed in Table 1. Of all the videos included, 58% (50/86) were categorized as pro, 22% (19/86) as anti, and 20% (17/86) as others. The number of days since upload ranged from 157 to 3331 days for proponent videos, 296 to 2437 days for opponent videos, and 563 to 4177 days for other videos. The highest view count was observed among the proponent videos, exceeding that of the opponent videos (Figure 1).

**Table 1.** Characteristics of the analyzed YouTube videos categorized by their stance on fluoride use (N=86) .

Characteristics	Pro	Anti	Other
Basic information			
Number of videos included	50	19	17
Days since upload			
Maximum	3331	2437	4177
Minimum	157	296	563
Average	1200	890	1465
Number of views			
Maximum	2,475,818	283,122	4,111,810
Minimum	91	986	16,731
Average	1,48,003	70,594	5,88,462
Views per day			
Maximum	1425	327	7303
Minimum	0	0	16
Average	147	104	748
Number of likes			
Maximum	13,319	4312	7671
Minimum	0	8	82
Average	969	1434	1781
Video length (s)			
Maximum	3616	1169	1247
Minimum	66	164	79
Average	597	624	541
Video creators			
Dentists and dental hygienists	30	1	13
Local government	2	0	0
Companies	2	0	0
Dental association	1	0	0
Layperson	15	16	4
Unknown	0	2	0
Target audience			
General public	49	19	17
Dental hygienists	1	0	0

In terms of the average daily views, calculated by dividing the total view count by the number of days since upload, 18% (9/50) of the proponent videos had fewer than 1 view per day, compared to only 5% (1/19) of the opponent videos and zero videos in the other sources. Conversely, videos with more than 19 views per day accounted for 44% (22/50) of the proponent videos, 94.7% (18/19) of the opponent videos, and 76.5% (13/17) of other videos.

In terms of the sources of the videos, 60% (30/50) of the proponent videos were uploaded by dentists or dental hygienists, 4% (2/50) videos by the local government, 4% (2/50) videos by companies, 2% (1/50) video by a dental association, and 30% (15/50) videos by laypersons. Of all the opponent videos, 5% (1/19) were uploaded by a dentist, 84% (16/19) by laypersons, and 11% (2/19) by unidentified sources.

Regarding the intended audience, 98% (49/50) of the proponent videos were directed at the general public, whereas 2% (1/50) video targeted dental hygienists. Conversely, 100% of the opponent videos were directed at the general public.

The first author (HSN) coded the mechanism of fluoride action, prevention, product introduction, evidence or guidelines, concentration, fluoride use, regular check-ups, safety confirmation, cost performance or cost-effectiveness, dietary habits, additives other than fluoride, dental implants, and trust relationships as the themes of the proponent video. In addition, HSN coded additives other than fluoride, product introduction, no confirmed safety, potential dangers, alternatives, and danger of Teflon coating as the themes of the opponent video.

Intercoder agreement was achieved in 332 of the 341 coding instances across the 23 (97.4%) selected videos. The Cohen κ statistic ranged from 0.91 to 0.95, with a mean of 0.93, indicating substantial intercoder reliability.

Table 2 shows a detailed breakdown of the themes addressed in the videos, frequency of discussion, and number and percentage of sources contributing to each category. This summary highlights the thematic focus and engagement of different sources by proponents, opponents, and others.

**Table 2.Table 2.** Code definitions and video source

Category	Theme	Examples	Frequency, n (%)	Sources, n (%)
Proponent				
Mechanism of fluoride action	Remineralization of enamel, Surface strengthening	Tooth remineralization, Strengthening the surface	33 (64)	Dentists and dental hygienists, 30 (91)
				Local government, 1 (3)
				Layperson, 2 (6)
Prevention	Fluoride involvement in caries prevention	Prevents tooth decay	24 (48)	Dentists and dental hygienists, 18 (75)
				Local government, 1 (4)
				Company, 2 (8)
				Dental association, 1 (4)
				Layperson, 2 (8)
Product introduction	Introduction of specific products	Check-Up and Systema (LION Dental Products Company Limited)	24 (48)	Dentists and dental hygienists, 21 (88)
				Company, 2 (8)
				Layperson, 1 (4)
Evidence/guidelines	Specific guidelines based on evidence	Evidence, Guidelines	24 (48)	Dentists and dental hygienists, 19 (79)
				Local government, 1 (4)
				Company, 2 (8)
				Dental association, 1 (4)
				Layperson, 1 (4)
Concentration	Fluoride toothpaste concentration based on age, high-concentration fluoride	1500 ppm for ages 6 and older	22 (44)	Dentists and dental hygienists, 16 (73)
				Local government, 1 (5)
				Company, 1 (4.5)
				Dental association, 1 (5)
				Layperson, 3 (14)
How to use fluoride	Amount of rinse water, usage per time, usage frequency per day	Rinse with a small amount of water, use toothpaste at least twice a day	16 (32)	Dentists and dental hygienists, 11 (69)
				Local government, 1 (6)
				Dental association, 1 (6)
				Layperson, 1 (6)
Regular check-ups	Regular check-ups at dental clinics	Let's go to regular check-ups at the dentist	13 (26)	Dentists and dental hygienists, 9 (69)
				Dental association, 1 (4)
				Layperson, 3 (23)
Safety confirmed	The safety of fluoride has been confirmed	Safety	10 (20)	Dentists and dental hygienists, 8 (80)
				Dental association, 1 (10)
				Layperson, 1 (10)
Cost performance/cost-effectiveness	Good cost performance for caries prevention, excellent cost-effectiveness	Fluoride mouthwash has good cost performance and is an effective prevention method	6 (12)	Dentists and dental hygienists, 3 (50)
				Company, 2 (33)
				Layperson, 1 (17)
Dietary habits	Reduce sugar intake, reduce snack frequency	Reduce sugar intake, reconsider the number of snacks	4 (8)	Dentists and dental hygienists, 1 (25)
				Layperson, 3 (75)
Additives other than fluoride	Sodium lauryl sulfate, triclosan, etc.	The foaming agent sodium lauryl sulfate is also found in detergents and is highly irritating	2 (4)	Dentists and dental hygienists, 2 (100)
Dental implants	Can also be used with dental implants	Commercial fluoride toothpaste can also be used with dental implants	2 (4)	Dentists and dental hygienists, 2 (100)
Trust relationship	Trust relationship between dentists and patients	The trust relationship between dentists and patients is important	1 (2)	Dentists and dental hygienists, 1 (100)
Opponent				
Additives other than fluoride	Additive-free, Free from additives	Additives are dangerous, strive for an additive-free lifestyle	14 (74)	Dentists and dental hygienists, 1 (7)
				Layperson 11 (79)
				Unknown, 2 (14)
Product introduction	Introduction of specific products	Shabondama Sekken Toothpaste (Shabondama Soap) and Pax Naturon (Taiyo Yushi Corp.)	10 (53)	Layperson, 8 (80)
				Unknown, 2 (20)
No safety confirmed	Fluoride is harmful, not safe	Considered dangerous by international organizations such as WHO, banned in some countries	7 (37)	Dentists and dental hygienists, 1 (14)
				Layperson, 5 (71)
				Unknown, 1 (14)
Potential dangers	Impedes child development, causes diseases	Impedes child development, causes Down syndrome, calcifies the pineal gland, etc	6 (32)	Layperson, 4 (67)
				Unknown, 2 (33)
Alternatives	Alternatives used when not using fluoride	Brushing with baking soda, salt, etc	6 (32)	Layperson, 6 (100)
Danger of Teflon coating	Dangers of Teflon-coated cookware	Teflon-coated frying pans are dangerous	2 (11)	Layperson, 2 (100)

a WHO: World Health Organization.

Among the proponent videos, the most-addressed category was the mechanism by which fluoride prevents caries, which was featured in 33 (64%) videos. Other frequently mentioned categories included information about the role of fluoride in caries prevention (24/50, 48%), specific product introductions (24/50, 48%), and evidence supporting the use of fluoride for caries prevention (24/50, 48%). Less commonly discussed categories included cost-effectiveness (n=6/50, 12%) and the trust relationship between dentists and patients (1/50, 2%). After completing the research, we translated all terms into English for the purpose of this report. Among the proponent sources, all videos uploaded by dentists and dental hygienists discussed the mechanism of action of fluoride, 63% (19/30) provided evidence for fluoride use in caries prevention, and 60% (18/30) provided information on the preventive role of fluoride. Videos from laypersons discussed categories such as regular dental checkups (3/15, 20%), dietary practices for caries prevention (3/15, 20%), and fluoride safety (1/15, 7%).

In the opponent videos, the most frequently addressed category was additives other than fluoride, which was mentioned in 14 (74%) videos. This was followed by specific product introductions (10/19, 53%) and claims regarding the lack of safety of fluoride (7/19, 37%). Alternatives to fluoride-containing toothpaste were discussed in 6 (32%) videos. A small number of videos (2/19, 11%) focused on the risks associated with nonfluoride substances, such as Teflon coatings, which were not associated with caries prevention. Among the opponent sources, laypersons frequently addressed additives other than fluoride (11/16, 69%), specific product introductions (8/16, 50%), and claims about the lack of safety of (7/16, 31%). Only 1 (5%) dentist discussed additives other than fluoride and alleged safety concerns regarding fluoride.

## Discussion

### Principal Findings

This study analyzed fluoride-related content on YouTube and categorized videos into proponent and opponent stances to evaluate the nature of the information being disseminated. Results revealed that the number of videos produced by proponents surpassed those produced by opponents, aligning with the trends identified in previous studies on fluoride-related content on Instagram [35]. However, previous studies analyzed the content of fluoride-related posts on YouTube and Instagram, which exhibited strong antifluoride tendencies [33,34,36]. Beyond differences in language and search timing, Japan’s sociocultural and policy context is also likely to have influenced these findings. Unlike many countries where community water fluoridation is implemented, Japan does not add fluoride to public water supplies [7]. As a result, caries prevention relies largely on individual and parental decisions (eg, whether to accept topical fluoride or purchase fluoride-containing products) rather than on passive, population-wide exposure. In this setting, messages portraying fluoride as unsafe may have a more direct impact on behavior. Moreover, because medical professionals in Japan traditionally hold strong authority in treatment decisions [16], statements made by dentists, including those expressing skepticism toward fluoride or endorsing holistic positions, may exert substantial influence even when not strongly evidence-based.

Proponent videos displayed a relatively high proportion (9/50, 18%) of content with fewer than 1 average daily view, compared to only 5% (1/19) for opponent videos, suggesting that opponent videos achieved more consistent viewership. Furthermore, 95% (18/19) of the opponent videos garnered more than 19 daily views, compared to only 44% (22/50) of proponent videos. This indicates that the opponent content may have reached a broader audience. Videos with over 1000 likes constituted 37% (7/19) of the opponent videos compared to 14% (7/50) of the proponent videos. Notably, 10% (5/50) of the proponent videos did not permit likes to be counted, potentially limiting their ability to visibly demonstrate audience support.

The content themes of proponent and opponent videos generally mirrored those identified in previous studies [30,32]. Most proponent videos, created predominantly by dental professionals (30/50, 60%), focused on themes such as the “mechanism of fluoride in preventing caries” (32/50, 64%), the “role of fluoride in caries prevention” (24/50, 48%), and “evidence-based guidelines” (24/50, 48%). These videos emphasize scientific and objective information. Among the extracted videos, almost all uploaded by dentists and dental hygienists mentioned the “mechanism of action of fluoride” (29/30, 97%). The caries prevention mechanism of fluoride is associated with various processes, including enamel remineralization and its effects on oral microorganisms, which are consistent with established facts regarding caries prevention [1]. This suggests that dental professionals produce persuasive content based on evidence and expertise. Studies indicate that expert-driven health communication is more effective in influencing individuals to adopt healthy behaviors [22-24]. However, the objective and fact-based nature of such content may limit its engagement potential for general audiences [20,21], thereby reducing its dissemination.

By contrast, videos by nonmedical individuals or organizations frequently highlighted themes such as dietary habits for caries prevention (3/15, 20%), the importance of regular dental checkups (3/15, 20%), and general claims regarding the safety of fluoride (1/15, 7%). These themes are often presented without supporting evidence, thereby reducing their credibility.

Opponent videos predominantly focused on concerns regarding “additives other than fluoride” (14/19, 74%), fluoride being “unsafe” (7/19, 37%), and fluoride being “dangerous” (6/19, 32%). These messages often align with broader preferences for natural or organic products and lifestyles that avoid synthetic additives [33]. Many of these videos also promoted specific fluoride-free products, subtly influencing consumer choice. Although excessive use of fluoride can lead to fluorosis [12], harmful effects are dose-dependent, and adverse outcomes are rare in typical use conditions [13]. Opponent videos that portray fluoride as “unsafe” or “dangerous” unnecessarily provoke fear, spread misinformation, and reinforce misconceptions.

In addition, 32% (6/19) of the opponent videos discussed alternatives to fluoride-containing products, providing practical guidance that may appeal to viewers. However, such content often serves as a marketing strategy to promote specific products [33,35]. Many topics mentioned in the videos were consistent with those in previous studies. However, new findings were observed in this study. Notably, 1 (15%) antifluoride video created by a dentist included content that not only opposed fluoride use but also criticized treatments involving metal prostheses, such as full metal crowns. This finding suggests a potential link between antifluoride sentiments and a broader skepticism toward established, evidence-based dental practices. Previous studies have stated insufficient evidence to support or refute the superiority of metal-free materials over metal-ceramics or other standard restorations for fixed prosthetic treatments [40]. Metal prostheses are a treatment option covered by public health insurance in Japan. Given the high level of trust placed on health care professionals, particularly in Japan, where medical decisions are often heavily influenced by doctors’ opinions [16], such videos may significantly influence the decision-making processes of patients.

Unlike previous studies, this study did not find any videos from either the pro- or antifluoride groups that mentioned water fluoridation or fluoride supplementation. This is likely because these fluoride application methods are not used in Japan [7]. Although easy access to health information brings many benefits, it also makes it easier for false, biased, or harmful content to spread [18]. Opponent videos often leverage sensational narratives to emphasize fluoride’s alleged risks, trigger strong emotions, and motivate viewers to share content or join discussions [20].

Social media platforms also enable the formation of echo chambers in which individuals with similar beliefs reinforce each other’s views, thereby further perpetuating misinformation [26]. The dissemination of such misconceptions can hinder the active use of fluoride by the general public, particularly in Japan, where there are no opportunities to receive passive fluoride application methods, such as water fluoridation. In addition, negative attitudes toward fluoride have led some parents to refuse fluoride treatment for their children [36], and these individuals often reject vaccinations [37]. This rejection of preventive health measures, fueled by misinformation on platforms such as YouTube, poses significant risks, potentially increases the prevalence of preventable diseases, escalates health care costs, and causes unnecessary suffering for individuals and families [38].

### Limitations and Strengths

This study is the first attempt at conducting a content analysis of Japanese YouTube videos addressing fluoride use for caries prevention, categorizing information into proponent and opponent perspectives. However, this study has several limitations.

First, it focused exclusively on Japanese YouTube videos, limiting the findings to information disseminated in Japanese and excluding perspectives from other cultural and linguistic contexts. Nevertheless, the results aligned with those of previous studies that analyzed fluoride-related social media content in other languages [35]. Second, our analysis was restricted to publicly available video sequences. YouTube also hosts “unlisted” videos, which are accessible only via direct links, and “private” videos, which are visible solely to individuals authorized by the uploader. As these visibility settings can change at any time, we excluded videos shared in closed or restricted communities. However, focusing on publicly accessible videos remains a significant endeavor, as they are most likely to appear in search results and reach a broad audience. Third, qualitative evaluation of video content may involve a degree of subjectivity. Although this study implemented a categorization process that required evaluative judgment, triangulation by multiple researchers was conducted to minimize bias and validate the findings. Finally, the findings are specific to the data collection period (October 15, 2023). Social media content is dynamic, and trends may shift over time or in response to current events, potentially leading to different patterns in other periods.

Despite these limitations, this study is noteworthy for its originality, as it offers a comprehensive content analysis of fluoride-related information on Japanese YouTube videos categorized by proponent and opponent perspectives.

### Conclusions

This study provides a comparative analysis of proponent and opponent content associated with fluoride use for caries prevention on YouTube. Proponent videos outnumbered opponent videos; the latter demonstrated a more consistent reach among viewers, achieving higher average daily views and a greater proportion of likes. Opponent videos relied on emotionally charged, sensational, and straightforward messages, such as “fluoride is harmful to the human body,” which provoked anxiety, encouraged empathy, and facilitated content sharing.

By contrast, proponent videos, predominantly produced by experts, such as dentists and dental hygienists, disseminate accurate, evidence-based information. However, these videos lack the emotional engagement necessary to capture the viewers’ attention and effectively foster resonance.

The potential consequences of such misinformation are significant. Parents opposing fluoride use have been shown to reject not only fluoride treatments for caries prevention but also vaccinations, reflecting a broader resistance to preventive health care measures. This could lead to an increased disease burden, higher healthcare costs, and unnecessary suffering from preventable illnesses among children and communities.

To counter these challenges, it is imperative to develop communication strategies that amplify the impact of proponent messages. These strategies should balance scientific rigor with accessible, practical, and engaging content that resonates with the viewers’ daily lives. Moreover, since antifluoride content often functions as a marketing strategy to promote specific fluoride-free products, it is imperative that communication strategies also counter these commercial motivations behind misinformation. Efforts must prioritize expanding the reach of accurate information to effectively combat the spread of misinformation and support public health initiatives.

## Supplementary material

10.2196/82265 Multimedia Appendix 1 Japanese keyword combinations used in the YouTube search.

10.2196/82265 Multimedia Appendix 2 Coding guidelines.
