# Supplementary material for: Pro- and Antifluoride Use Messages on YouTube in Japan: Content Analysis
Source: JMIR Form Res. 2025 Dec 29;9:e82265. doi: 10.2196/82265 (PMC12747662; doi:10.2196/82265)
Supplement: Multimedia Appendix 1 [file formative-v9-e82265-s001.docx]

| **Appendix 2. Japanese keyword combinations used in the YouTube search** | |
| --- | --- |
| Japanese | English |
| フッ素　コーティング | fluoride AND coating |
| フッ素　加工 | fluoride AND processing |
| 歯医者　フッ素 | dentist AND fluoride |
| フッ素　コート | fluoride AND coat |
| フッ素　歯磨き粉 | fluoride AND toothpaste |
| フッ素　加工　フライパン | fluoride AND pan coating |
| フッ素　樹脂 | fluoride AND resin |
| フッ素　危険 | fluoride AND danger |
| フッ素　うがい | fluoride AND rinse |
| ふっそ | fluoride |
