# Supplementary material for: Pro- and Antifluoride Use Messages on YouTube in Japan: Content Analysis
Source: JMIR Form Res. 2025 Dec 29;9:e82265. doi: 10.2196/82265 (PMC12747662; doi:10.2196/82265)
Supplement: Multimedia Appendix 2 [file formative-v9-e82265-s002.docx]

| **Appendix 1. Coding Guidelines** | |
| --- | --- |
| Pros | The video concludes that fluoride use is advisable. Even in the absence of a clear conclusion, the content evidently advocates for fluoride use. |
| Cons | The video concludes that fluoride use is inadvisable. Even in the absence of a clear conclusion, the content evidently opposes fluoride use or asserts its lack of necessity. |
| Other | The video presents both pro- and anti-fluoride viewpoints (e.g., highlighting both benefits and risks, or suggesting that fluoride is necessary for children but not for adults). |
